# Mapping the global potential transmission hotspots for severe fever with thrombocytopenia syndrome by machine learning methods

**DOI:** 10.1080/22221751.2020.1748521

**Published:** 2020-04-29

**Authors:** Dong Miao, Ke Dai, Guo-Ping Zhao, Xin-Lou Li, Wen-Qiang Shi, Jiu Song Zhang, Yang Yang, Wei Liu, Li-Qun Fang

**Affiliations:** a State Key Laboratory of Pathogen and Biosecurity, Beijing Institute of Microbiology and Epidemiology, Beijing, People’s Republic of China; b Department of Biostatistics, College of Public Health and Health Professions, and Emerging Pathogens Institute, University of Florida, Gainesville, FL, USA

**Keywords:** Severe fever with thrombocytopenia syndrome, *Haemaphysalis longicornis*, machine learning, modelling, distribution, risk assessment, world

## Abstract

Severe fever with thrombocytopenia syndrome (SFTS) is an emerging infectious disease with increasing spread. Currently SFTS transmission has expanded beyond Asian countries, however, with definitive global extents and risk patterns remained obscure. Here we established an exhaustive database that included globally reported locations of human SFTS cases and the competent vector, *Haemaphysalis longicornis* (*H. longicornis*), as well as the explanatory environmental variables, based on which, the potential geographic range of *H. longicornis* and risk areas for SFTS were mapped by applying two machine learning methods. Ten predictors were identified contributing to global distribution for *H. longicornis* with relative contribution ≥1%. Outside contemporary known distribution, we predict high receptivity to *H. longicornis* across two continents, including northeastern USA, New Zealand, parts of Australia, and several Pacific islands. Eight key drivers of SFTS cases occurrence were identified, including elevation, predicted probability of *H. longicornis* presence, two temperature-related factors, two precipitation-related factors, the richness of mammals and percentage coverage of water bodies. The globally model-predicted risk map of human SFTS occurrence was created and validated effective for discriminating the actual affected and unaffected areas (median predictive probability 0.74 vs. 0.04, *P* < 0.001) in three countries with reported cases outside China. The high-risk areas (probability ≥50%) were predicted mainly in east-central China, most parts of the Korean peninsula and southern Japan, and northern New Zealand. Our findings highlight areas where an intensive vigilance for potential SFTS spread or invasion events should be advocated, owing to their high receptibility to *H. longicornis* distribution.

## Introduction

Severe fever with thrombocytopenia syndrome (SFTS) is an emerging tick-borne infectious disease caused by a novel bunyavirus (SFTSV), with a wide clinical spectrum, ranging from mild febrile disease accompanied by thrombocytopenia and/or leukocytopenia to severe hemorrhagic fever, clinical encephalitis or multiple organ failure, with a case fatality rate (CFR) of 12‒50% [1,2]. The disease was first identified in China in 2009 but retrospectively traced back to human cases in 2007 [3,4]. South Korea and Japan both reported their first cases in 2013, and were traced retrospectively to 2010 in South Korea [5–7]. Tick-to-human transmission is the primary route by which people are infected with SFTSV, and *Haemaphysalis longicornis* (*H. longicornis*) ticks act as the main transmission vector [8–10]. *H. longicornis* is native to East Asia and has established populations in the Australasian and Western Pacific Regions [11–13]. Very recently, this tick species has been reported in the United States, first observed to infest the sheep in New Jersey and then identified in seven states and the suburb of New York City [14,15]. The recent disease reports in wider Asia range [16,17], together with the trans-regional dispersion of the competent vector further warned a high possibility of increasing epidemic of SFTS in the future. The most recent efforts to identify populations at risk have been proposed based on the distribution of *H. longicornis*, however lacking model-based prediction and not at international level, thus the spatiotemporal distribution pattern of the disease in the worldwide range warranted intense investigation.

In this study, we adopted a two-step strategy to predict the potential distribution of SFTS. For the first modelling step, the global distribution of *H. longicornis* was predicted based on data regarding the known locations of its occurrences by using the ecological niche (EN) modelling approach, which has been widely used in modelling species distribution when only presence data is available for prediction [18,19]. At second step, potential hotspots of human SFTS occurrence in the world were predicted, by establishing a decent boosted regression tree (BRT) model that used SFTS infection data in China and a range of environmental, climate and biological covariates. BRT model has been widely applied in modelling risk for infectious disease, e.g. avian influenza A (H7N9) and ZIKV disease etc., because it performs well in predicting distributions of organisms while accounting for non-linear relationships and interactions between the risk and covariates [20–22]. This information might offer epidemiologists, parasitologists and veterinaries about potential spread of SFTSV, thus to attain a targeted surveillance in predicted risk areas, and lay foundation for understanding other tick-borne pathogens dispersion across continents.

## Materials and methods

### Data collection and management

A comprehensive database was established by combining all SFTS case report, the recorded geographic distribution of *H. longicornis*, and a set of 56 explanatory variables at the county level in the world. Briefly, the report data of SFTS patients in China was extracted from the China Information System for Diseases Control and Prevention (CISDCP). The data on the SFTS patients in South Korea and Japan were obtained from the Korea Centers for Disease Control and Prevention (KCDC) (https://www.cdc.go.kr/) and the National Institute of Infectious Diseases (NIID) of Japan (https://www.niid.go.jp/), both supplemented by data in the published literature [16]. For each reported SFTS cases during January 2010–December 2018, the demographic information and the geographic information at the county level were obtained. The imported cases were georeferenced according to the site of acquiring infection, instead of their usual residence. A comprehensive dataset of globally reported presence of *H. longicornis* was established that included data recording on survey sites and positive locations of *H. longicornis* in latitude and longitude, as well as the survey dates on record (if no survey date is available, the publication date of the literature is used instead) (Supplementary Dataset S1) [15,23–26]. The 56 explanatory covariates mainly comprised data regarding eco-geography, climate, environment, mammalian richness and areas of migration and habitat of migratory birds on global range, which were chosen due to their potential effect on the suitability for SFTS transmission based upon the previous studies [27,28]. Eco-geographical and climatic variables including global average climate data “WorldClim” during 1950–2000, elevation, population density, livestock density, land cover and mammalian richness were respectively collected and extracted at a resolution of 0.1° × 0.1° grid as well as at the administrative area level (mostly county level) for assessment and prediction of the global distribution of *H. longicornis* [29–34]. The Global Mammal Richness Grids data set can provide information at a one-kilometer spatial resolution with grid cell values represent the number of species in a particular class, family, or threatened category issued by International Union for Conservation of Nature (IUCN) [34]. Data on the migration and habitat of migratory birds were provided by the BirdLife International [35].

### Modelling risk for H. longicornis distribution

Each occurrence record of *H. longicornis* was geo-referenced and linked to the digital world map according to its coordinates using Geographic Information System (GIS) technologies. Machine learning method with ecological niche (EN) model was applied at the 0.1° × 0.1°grid level to correlate the global observed locations of *H. longicornis* and 51 selected eco-geographical and climatic variables (Supplementary Table S1). The grid locations with the occurrence of *H. longicornis* were used in the model. Ten thousand grids were randomly selected from all grids without the presence of *H. longicornis* as control points for the model. The variables were first grouped according to the subject (e.g. eco-climatic, land cover), and then multicollinearity was screened for each group and only one variable was chosen as the proxy of highly correlated variables (correlation coefficient ≥0.7) to be used for model-fitting (Supplementary Tables S2–S4). The variables whose contribution was less than 1% were removed from the ecological niche (EN) model (Supplementary Table S5). Ultimately, 10 variables were identified for the EN model after the variable selection, including elevation, three eco-climatic variables, three land cover types, population density, livestock density, and mammalian richness. In this study, the maximum entropy method (Maxent) was used [18,19,36], and the model-predicted probabilities of the *H. longicornis* presence were mapped to display the potential habitat suitable for this vector tick around the world [19]. Technical details regarding model formulation and data inclusion and exclusion criteria are given in Supplementary Methods.

### Modelling risk for SFTS occurrence

A boosted regression tree (BRT) approach was used to establish a multivariate empirical relationship between the probability of SFTS presence and the eco-geographic and environmental conditions at the county level (GADM database, version 2.8) as had been widely used for assessing risk of infectious diseases [20–22,37–39]. The “case–control” design at the county level was used for the analysis, briefly, all counties with SFTS cases were used as “case units,” and a three-fold sampling of 1116 counties without SFTS cases were randomly chosen as “control units,” where all units were limited to China. When considering the inclusion of variable types, we simplified and integrated the specific land cover types, and the other variables included bio-climatic, population and livestock density, mammalian richness and elevation, predicted probability of *H. longicornis* presence (Supplementary Table S1). We adopted the same variable selection method as the EN model, accounting for non-linear association and interactions between covariates (Supplementary Table S2, Table S4 and Table S6), the variables whose contribution was less than 1% were eliminated to screen out the high-efficiency predictive variables (Supplementary Table S7). Finally, for each county, altogether 18 independent variables were considered in the model, which included 5 bioclimatic variables determined from 1950 to 2000, density of population, elevation, percentage coverages of land cover (irrigated croplands, rainfed croplands, needleleaved forests, broadleaved forests, other forests, grassland, build-up land and water bodies), the density of livestock, mammalian richness, predicted probability of *H. longicornis* presence which was extracted from the ecological niche model. A bootstrapping procedure was conducted for the BRT models to generate robust estimates of predicted probability, based on which the mean transmission risk of SFTS around the world was predicted (Supplementary Methods). The BRT models were also validated to be effective for discriminating the actual affected and unaffected areas by using the data in three SFTS-reporting countries outside China, i.e. Japan, South Korea and Vietnam.

## Results

### The recording of SFTS cases on the global range

**Figure 1.**
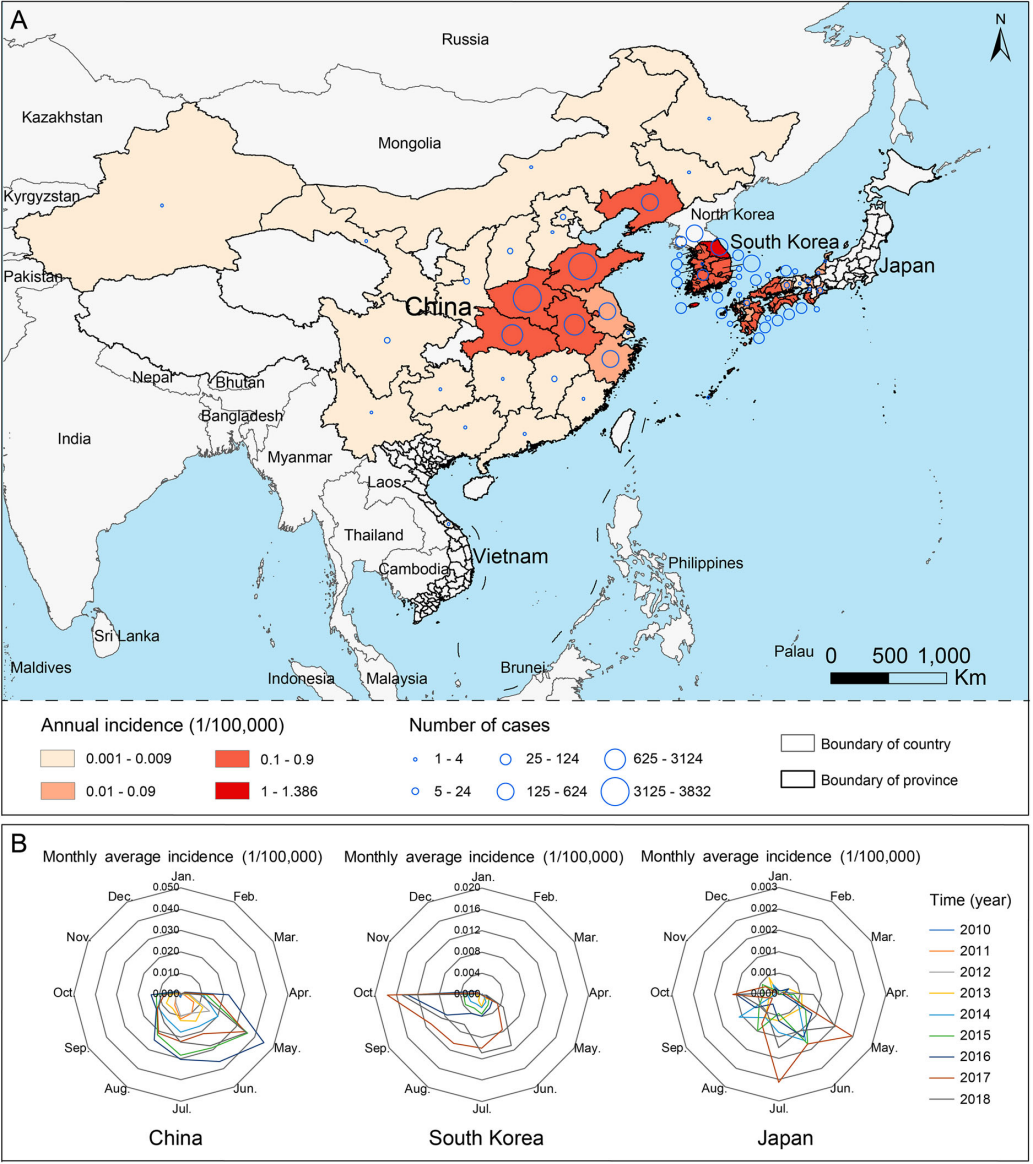
The globally spatial and seasonal distributions of SFTS during 2010–2018. (A) The average annual incidence and the number of SFTS cases were indicated at the provincial level. (B) The seasonality is presented as a radar diagram for each of three mainly affected countries by SFTS, including China, Japan and South Korea. The circumference is divided into 12 months in a clockwise direction, and the radius represents average monthly incidences over 2010–2018.

As of 31 December 2018, a total of 13,259 SFTS patients were recorded, including 11,995, 396, and 866 cases who were respectively reported from China, Japan and South Korea, as well as 2 cases retrospectively identified in Vietnam. Of all SFTS cases, 1161 died, leading to an overall case fatality rate (CFR) of 8.76%, with the highest CFR observed in Japan (27.02%, 107/396). The age and gender pattern differed significantly among the three major affected countries, with patients in China having the lowest median age (62 years old), and highest female proportion (53.15%) (Table 1). Over the surveillance years, the annual incidence obviously increased with the average annual percent change (AAPC) estimated as 21.90%, 12.20%, and 53.07% in China, Japan, and South Korea, respectively. The number of affected provincial or administrative regions was 25, 23 and 17 in China, Japan and South Korea, respectively. In two countries, the reported cases were geographically concentrated, basically in the eastern and central China and the southern Japan, but distributed throughout South Korea (Figure 1A). The disease epidemic spanned from May to July in China and Japan, and from June to October in South Korea (Figure 1B).

**Table 1.** Baseline demographic characteristics of reported SFTS patients in China, Japan and South Korea from 2010 to 2018.

	China	Japan	South Korea
No. of reported cases	11,995	396	866
Annual incidence (/10^5^)	0.10	0.04	0.19
Average annual percent change (95% CI)	21.90% (−5.49%–49.29%)	12.20% (−20.06%–44.46%)	53.07% (2.30%–103.84%)
No. of deaths	881	107	173
Case fatality rate (%)	7.34	27.02	19.98
Age median (IQR)**	62 (52–70)	74 (5–96)	65 (64–66)
No. (%) of female cases	6375 (53.15%)	204 (51.52%)	436 (50.35%)
Major epidemic seasons	May–July	May–July	July, September–October
No. of affected provinces	25	23	17
Top 5 affected Provinces (total case number)			
Top 1	Henan (3832)	Miyazaki (61)	Gyeonggi-do (146)
Top 2	Shandong (3466)	Kagoshima (39)	Gyeongsangbuk-do (136)
Top 3	Anhui (1833)	Yamaguchi (37)	Gangwon-do (125)
Top 4	Hubei (1566)	Hiroshima (35)	Gyeongsangnam-do (79)
Top 5	Liaoning (561)	Kochi (34)	Chungcheongnam-do (70)

### The global distribution and environmental suitability of H. longicornis tick

**Figure 2.**
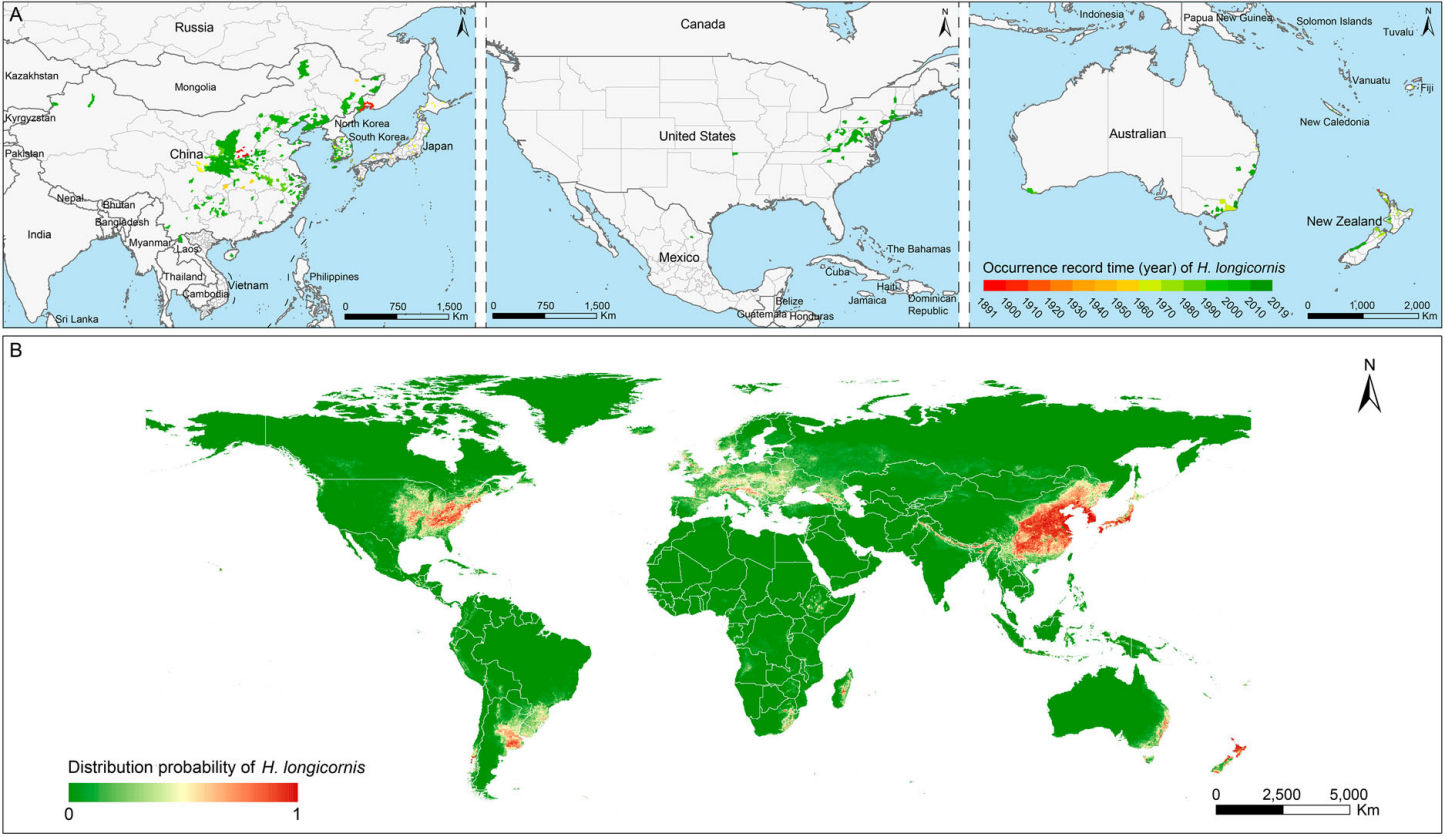
Global recorded locations and model-predicted distribution probability of *H. longicornis*. (A) Each occurrence record of *H. longicornis* was geo-referenced and linked to the digital world map. The recording time of the *H. longicornis* presence was marked by colour gradients from red to green. (B) The potential geographic range of *H. longicornis* was predicted and mapped by using a maximum entropy method based on eco-geographical and climatic variables.

A thematic map was created to display the geographic dispersion dynamics of *H. longicornis* on the global range (Figure 2A). The *H. longicornis* tick was first reported in central China and eastern Russia in 1891, subsequently recorded from New Zealand and Japan in 1910s, from South Korea, and Australia in 1960s, with the most recent report in 2010s that was observed in the USA. Based on the data of all observed locations of *H. longicornis* presence, the global environmental suitability for *H. longicornis* was determined by 10 global variables, with each of their relative contribution (RC) ≥1% to the established ENM model. Among which the precipitation of warmest quarter was the leading factor that contributed to the probability of *H. longicornis* presence, with the greatest RC of nearly 33%, followed by annual mean temperature, density of population, and density of livestock, with all their RCs exceeding 5% (Table 2). Other six variables, including mammalian richness, herbaceous vegetation of closed to open type, mean diurnal range of temperature, rainfed croplands, elevation, broadleaved deciduous forest of closed type, contributed moderately to the probability of *H. longicornis* presence with their RCs ranging between 1 and 3 (Table 2). The model has shown decent predictive power, yielding a mean area under curve (AUC) being 0.967 ± 0.001 (Supplementary Figure S1). The exact associations between the probability of *H. longicornis* presence and the four main drivers with RC ≥5% were shown in Supplementary Figure S2.

**Table 2.** The contribution of environmental variables to predict the global risk probability of *H. longicornis* presence based on ecological niche model.

Variable	Relative contribution	
	Mean	Sd
Precipitation of warmest quarter (mm)	33.03	2.07
Annual average temperature (°C)	27.90	2.54
Population density (1 person/km^2^)	19.68	3.57
Livestock density (1 head/km^2^)	9.32	3.19
Mammalian richness (1 species/km^2^)	2.89	0.84
Percentage of herbaceous vegetation of closed to open type (%)	1.98	0.63
Mean diurnal range of temperature (°C)	1.61	1.14
Rainfed croplands (%)	1.28	1.10
Elevation (m)	1.20	0.57
Percentage of broadleaved deciduous forest of closed type (%)	1.11	0.36

Based on the ENM-predicted probability, global distribution of *H. longicornis* presence was mapped (Figure 2B). The potential habitat suitable for this vector tick were significantly wider than the already reported areas, mainly distributed in the eastern and central China, the southeast Russia that bordered China and North Korea, the whole country of North Korea, South Korea and Japan, the eastern coastal areas of Australia, the southwestern coastal and northern regions of New Zealand and several Oceania countries/regions. It is notable that a range of areas in the eastern and central USA were also highly suitable for the *H. longicornis* ticks propagation, significantly wider than the already reported areas. In addition, the southeastern and southwestern coastal areas of South America, southern Europe, the southeastern coastal areas of Africa and Madagascar, and the northern area of western Asia also showed a high environmentally suitability for *H. longicornis* tick.

### The global distribution and potential ecological-risk area of human SFTS

**Figure 3.**
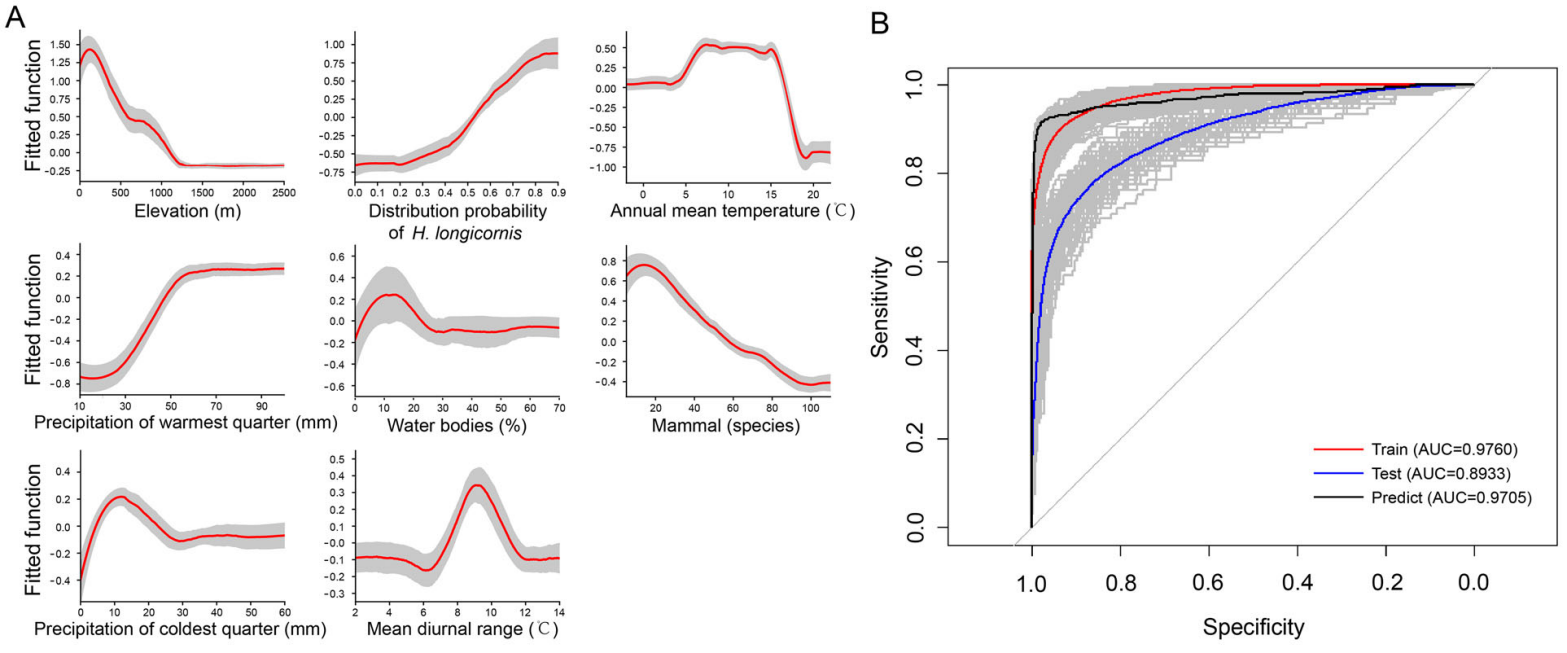
ROC curves for the risk probability of SFTS transmission based on climatic, eco-geographical and social variables by using BRT models. (A) The red curves are average predicted lines for risk factors by 100 repeats (grey lines) based on the bootstrapping procedure. (B) The grey lines are the ROC curve for 100 repeats, and the red, blue and black lines indicate the average ROC curves of 100 repeats based on the bootstrapping procedure for the train set, test set and prediction, respectively.

According to the BRT models created by using the distribution of SFTS cases in China, 8 significant variables with RCs greater than 5% were shown to be important drivers for the presence of SFTS cases, including elevation, predicted probability of *H. longicornis* presence, annual mean temperature, precipitation of warmest quarter, percentage of water bodies, mammalian richness, precipitation of coldest quarter, mean diurnal range of temperature, with descending order for their effect size (Table 3). Among the significant contributors, elevation had the highest RC of about 17.3%, with a negative effect on the SFTS presence, for example, a higher risk of the disease presence was seen in regions where the average elevation was less than 1000 m (Figure 3A). Higher risk of SFTS presence was positively associated with the model-predicted probability of *H. longicornis* occurrence, with RC as high as 11.8%. Two temperature-related factors contributed to the risk of SFTS presence with quadratic effect, for example, a higher risk was related to the annual mean temperature between 5°C and 15°C and the mean diurnal range between 8°C and 11°C. Two precipitation-related factors were positively associated with higher risk of SFTS presence, including precipitation in the warmest quarter and driest month. The richness of mammals was positively related to the risk of SFTS presence in regions hosting <20 mammals species, while negatively related to the SFTS risk in regions hosting ≥20 mammals species. The percentage coverage of water bodies had a quadratic effect on the risk of SFTS presence. There were other seven variables which contributed to the presence of SFTS cases with moderate effects, including the percentage coverage of irrigated croplands, other forests, needleleaved forests and broadleaved forests, together with precipitation of driest month, livestock density and population density with all their RCs ranging from 2% to 5% (Table 3).

**Table 3.** The contribution of environmental variables to predict the occurrence of SFTS cases on the global range based on boosted regression trees model.

Variable	Relative contribution	
	Mean	Sd
Elevation (m)	17.32	2.29
Predicted probability of *H. longicornis* presence	11.77	2.12
Annual mean temperature (°C)	11.58	1.49
Precipitation of warmest quarter (mm)	8.12	2.02
Water bodies (%)	6.75	1.81
Mammalian richness (1 species/km^2^)	6.19	1.03
Precipitation of coldest quarter (mm)	5.57	1.63
Mean diurnal range of temperature (°C)	5.41	1.57
Irrigated croplands (%)	4.87	1.45
Precipitation of driest month (mm)	4.56	1.81
Other forests (%)	3.25	1.02
Livestock density (1 head/km^2^)	2.65	0.74
Population density (1 person/km^2^)	2.22	0.69
Needleleaved forests (%)	2.13	0.74
Broadleaved forests (%)	2.12	0.75
Build-up land (%)	1.95	0.57
Rainfed croplands (%)	1.92	0.50
Grassland (%)	1.62	0.46

**Figure 4.**
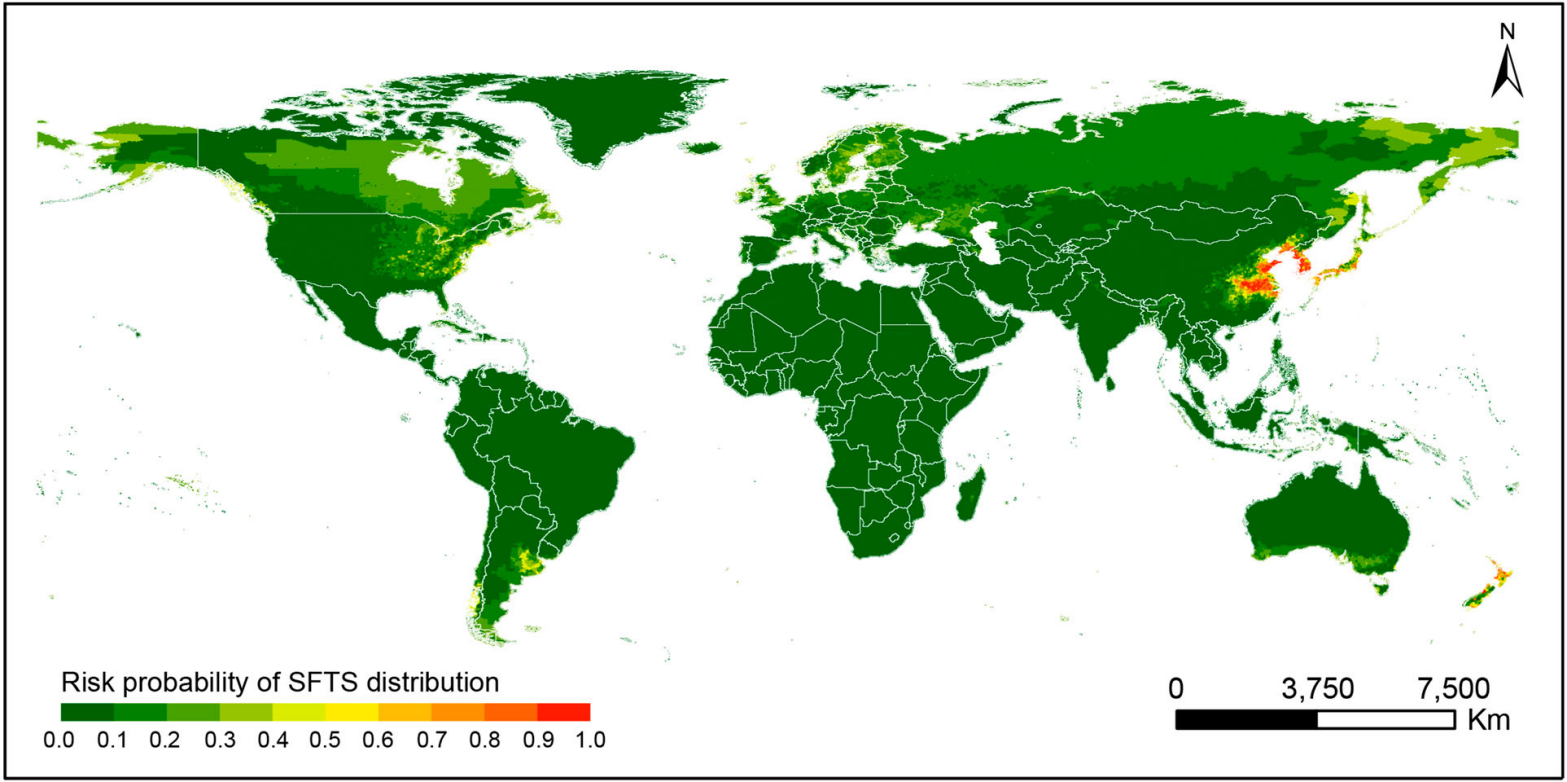
The potential distribution of human SFTS on global range based on BRT model. The probability of SFTS transmission was marked by different colour blocks.

The predictive map for occurrence of SFTS was created, demonstrating the highest-risk areas located in the east-central China, in most parts of the Korean peninsula and southern Japan, additionally at the coastal areas in northern and limited region in the southern New Zealand (Figure 4). The receiver-operating characteristic (ROC) curve was produced for the BRT models and area under the curve (AUC) was calculated to be 0.971 (95% CI: 0.964–0.977), indicating an excellent prediction power for emergence of human SFTS cases (Figure 3B). The overall predictive performance is satisfactory with AUCs of 0.976, 95% CI: 0.975–0.977 for the training datasets and 0.893, 95% CI: 0.889–0.897 for the testing datasets respectively. The goodness of fit of the BRT models was evaluated using the Hosmer-Lemeshow test, showing a decent risk discrimination between counties with and without SFTS cases (median of *X*^2^ = 11.72, *P*-value = 0.38). To avoid overconfidence in the model, we also performed a sensitivity analysis by using sampling at the county level, namely the controls were set as the no-case-reporting-counties within 25 provinces that reported SFTS cases in China. The ROC and the AUC’s are similar to our current model that samples the whole country (the estimated AUC value of 0.971, 95%CI: 0.965–0.978). The model-predicted risk probability of SFTS presence was further validated by using the reported data in three SFTS-affected countries, Japan, South Korea and Vietnam. The results showed effective for discriminating the actual affected and unaffected areas (*P* < 0.001), with a median predictive value of 0.74 (IQR: 0.54–0.86) for affected counties and 0.04 (IQR: 0.03–0.05) for unaffected counties.

## Discussion

Since its discovery in 2010, over 13 thousands reported cases of SFTS have been documented worldwide and more than 8% of those cases have died. There is a significantly increasing trends in all the three mainly affected countries, with above 90% cases occurred in China, while the highest annual increasing rate observed in South Korea, and the highest case fatality rate observed in Japan, which indicates a serious public health concerns, given that no effective treatment or vaccine is available at this moment [40]. Recent detection of *H. longicornis*, and its quick spread in the United State demonstrated the possibility of the disease to be global rather than regional [15]. In this study, we predicted a high receptivity to *H. longicornis* across two continents, including northeastern USA, New Zealand, parts of Australia, and several Pacific islands (New Caledonia, Fiji, Western Samoa, Tonga, and Vanuatu). Our prediction map (Figure 2) revealed seven countries needed intensive surveillance for *H. longicornis* presence, while there were fifteen countries which had only small and sparse risky areas that needed moderate surveillance. A special situation was observed in the New Zealand, which had reported the presence of *H. longicornis* tick since 1910s, however, no SFTS cases had been reported until now. According to the phylogenetic study in China, SFTSV was suggested to originate 50–150 years ago, and the viral population only experienced a recent growth phase, with the first human infection reported in 2009 in China [41]. The SFTSV viral lineage, if already existed in New Zealand, might remained unknown to cause human infection without adequate sampling and tests. On the other hand, the long distance between the New Zealand and Asian countries, together with less population travel or commercial interchange than with the neighbouring countries might lead to the less likelihood of introducing the viral in a short period. All these assumptions call for the necessity of establishing a wide-scope surveillance of SFTSV in this country. Recently another emerging SFTSV-like bunyaviruses, Heartland virus (HRTV) that caused similar severe febrile disease and death in humans was isolated from patients in the United States [42–44]. In case the HRTV could be also harboured and transmitted by *H. longicornis*, the possibility of local transmission of HRTV in North America should not be neglected. On the other hand, based on the current BRT Model, the predicted distribution of *H. longicornis* contributed significantly to the SFTS occurrence, further corroborating the vital role of this tick in maintaining and transmitting the disease in natural environment. An intensive vigilance for potential SFTS spread or invasion events should be advocated in the *H. longicornis* receptible countries [45].

When two models were considered together, eight significant variables overlapped for predicting both the distribution of the *H. longicornis* and SFTS cases, including elevation, precipitation of warmest quarter, annual mean temperature, mean diurnal range of temperature, livestock density, mammalian richness, population density and rainfed croplands, although different relative contributions of them were found in restraining their distributions. These factors are generally reflective of the environments where wild and/or domestic animal hosts exist and enable tick survival, virus circulation and adequate exposure of human hosts to the virus [46,47].

There are additional factors that contribute to the potential distribution of human cases, including irrigated croplands, build-up land, grassland and percentage of water bodies, which were considered as important driver for the risk of human infection, probably by affecting the human behaviour. Percentage of water bodies, on the other hand, was considered as a potential factor in affecting the viral transmission to a specific region by the long-distance flying of the migratory birds, as had been previously hypothesized [48].

Among these significant factors contributing to the human infection risk, elevation, temperature and precipitation indices have been found to be important drivers of SFTS infection in the past studies [27]; Strong correlations have been found in Henan Province of central China between SFTS risk and suitable environments for ticks, including grass and shrub cover, as well as forested land fragmented by agricultural or shrub cover [49]. Non-irrigated agricultural land cover has also been found to be associated with SFTS incidence in Hubei province in China [50]. These findings corroborated the important role of these factors by independent studies.

For the first time, we have strived to be exhaustive in the assembly of contemporary data on SFTS occurrence, the eco-climatic and environmental factors. On top of that, we have applied new modelling approaches to maximize the predictive power of these data. In comparison with previous efforts to map the distribution of *H. longicornis* [24], the current modelling efforts had been remarkably improved by fitting with the most recent occurrence data and with the given complex covariates, e.g. the density of livestock and richness of mammals. These advantages enabled a better identification of at-risk areas by using environmental correlations that were determined in areas with intensive SFTS surveillance efforts.

In recent years, tick-borne phleboviruses (TBPVs) have attracted much attention because of their increasing incidence. In addition to SFTSV and HRTV which had severely threatened human health, other novel TBPV continued to be identified and isolated from ticks, such as Guertu virus (GTV) from *Dermacentor nuttalli* ticks in Xinjiang Province, China [51], and Hunter island group virus (HIGV) isolated from ticks in Australia [52]. In addition to SFTSV, *H. longicornis* also transmits *Bartonella*, *Ehrlichia*, *Anaplasma*, *Borrelia* spp., which cause human infection [53,54]. The current model developed for *H. longicornis* and SFTS prediction could lend useful strategy for other TBPVs and other tick borne agents with similar ecological niche.

Limitations of this study are needed to be recognized that are relevant to the interpretation of the results. First, because of the inaccuracies in human case report data, especially in the low-risk region, the lack of valid diagnosis methods and low vigilance to the disease of the local public health were responsible for the underestimation of the human cases, which might lead to the misclassification of the regions with or without risk of SFTS occurrence. Second, some potential determinants of the human SFTSV infections were not included in our BRT model due to the lacking of data. This included but not limited to density of the main vector tick (*H. longicornis*), the infection rate of SFTSV in *H. longicornis* and animal hosts, which would inevitably result in a bias in the risk evaluation. All these limitations called for a further systematic epidemiological study that fully consider the complex interaction between SFTSV, the competent transmission tick species, their hosts, and the habitat environment after obtaining a continuous and evenly sampling and detection of SFTSV by field researches. Still, this study represented the first mapping of the potential-risk areas of SFTS occurrence at the global level. The high resolution risk maps provided an evidence base to prioritize areas for targeting risk zones, where public health authorities should be most vigilant for potential spread or importation events, and advocate intensive surveillance on *H. longicornis* population and the situation of SFTSV infection.

## Supplementary Material

Supplemental Material
